# *In vivo* active-targeting fluorescence molecular imaging with adaptive background fluorescence subtraction

**DOI:** 10.3389/fonc.2023.1130155

**Published:** 2023-03-14

**Authors:** Jorge D. Vega, Daiki Hara, Ryder M. Schmidt, Marwan B. Abuhaija, Wensi Tao, Nesrin Dogan, Alan Pollack, John C. Ford, Junwei Shi

**Affiliations:** ^1^ Department of Radiation Oncology, Miller School of Medicine, University of Miami, Miami, FL, United States; ^2^ Department of Biomedical Engineering, University of Miami, Coral Gables, FL, United States

**Keywords:** fluorescence molecular imaging, fluorescence molecular tomography, nanoparticle imaging, active targeting, fluorescence background subtraction, multi-modality imaging, contrast CT, image-guided irradiation

## Abstract

Using active tumor-targeting nanoparticles, fluorescence imaging can provide highly sensitive and specific tumor detection, and precisely guide radiation in translational radiotherapy study. However, the inevitable presence of non-specific nanoparticle uptake throughout the body can result in high levels of heterogeneous background fluorescence, which limits the detection sensitivity of fluorescence imaging and further complicates the early detection of small cancers. In this study, background fluorescence emanating from the baseline fluorophores was estimated from the distribution of excitation light transmitting through tissues, by using linear mean square error estimation. An adaptive masked-based background subtraction strategy was then implemented to selectively refine the background fluorescence subtraction. First, an *in vivo* experiment was performed on a mouse intratumorally injected with passively targeted fluorescent nanoparticles, to validate the reliability and robustness of the proposed method in a stringent situation wherein the target fluorescence was overlapped with the strong background. Then, we conducted *in vivo* studies on 10 mice which were inoculated with orthotopic breast tumors and intravenously injected with actively targeted fluorescent nanoparticles. Results demonstrated that active targeting combined with the proposed background subtraction method synergistically increased the accuracy of fluorescence molecular imaging, affording sensitive tumor detection.

## 1 Introduction

Employing fluorescent nanoparticles (FNPs), fluorescence molecular imaging (FMI) particularly fluorescence molecular tomography (FMT) can noninvasively identify the molecular processes of interest and has been widely applied in pre-/co-clinical studies in drug development, disease detection, screening, diagnosis, and treatment evaluation (1–4). As a three-dimensional (3D) molecular imaging modality, FMT demonstrates its distinct performance and wide applications in oncology studies, due to its advantages of low cost, high throughput, high specificity, and high sensitivity (5–8). For example, we previously developed a small animal multimodality imaging and radiotherapy platform (SAMMI-RT), by integrating FMT, x-ray computed tomography (CT) and radiation treatment (9). In this platform, FMT can not only specifically identify tumor to guide radiation delivery, but also assess tumor progression and treatment response at molecular level. What is more, FMT introduces no extra ionizing radiation to the image-guided radiotherapy studies.

Compared to conventional fluorescent dyes, FNPs demonstrate stronger fluorescent brightness, better photostability, water dispersibility, and biocompatibility, affording high-sensitive *in vivo* FMI/FMT (10, 11). Moreover, thanks to its large surface area and easy modification, FNP provides a platform for the design of smart theranostic probe for tumor-targeted diagnosis and treatment (12, 13). Actively targeted FNPs have been widely investigated for their potentials in enhancing specificity and sensitivity for cancer imaging and treatment response assessment (14–18). Two distinguishing paths have been explored by 1) targeting to cancer cells due to the overexpression of transferrin, folate, epidermal growth factor, or glycoproteins etc. and 2) targeting to the tumor endothelium due to the overexpression of the vascular endothelial growth factors (VEGF), αvβ3 integrins, the vascular cell adhesion molecule-1 (VCAM-1) or matrix metalloproteinases (MMP) etc. (11, 19). For example, to selectively target breast tumors overexpressing Epidermal Growth Factor Receptors (EGFR), we also developed the actively-targeted FNP by conjugating fluorescent poly lactic-co-glycolic acid (PLGA) nanospheres with anti-EGFR antibodies (9). Although the developed PLGA-anti-EGFR FNPs could accumulate in the targeted tumor with enhanced concentration, there still exists nonspecific uptake of nanoparticles throughout the animal body. Together with the intrinsic autofluorescence from the endogenous chromophores (20), these nonspecific background signals will mix with the tumor fluorescence signal, therefore reducing tumor-detection sensitivity and specificity, particularly when the tumor signal is weak.

An effective background reduction strategy is crucial to improve the tumor detection sensitivity in *in vivo* FMI and is particularly useful when exogenous nanoparticles are used as imaging probes. Take the PLGA-anti-EGFR nanoparticles employed in this study as an example, there are several situations wherein an effective background reduction method can be especially helpful: 1) tumor is small; 2) tumor targeting is compromised due to previous EGFR-targeting treatments; 3) a considerable number of nanoparticles have been cleared out of the tumor volume. All these situations stand for the weak tumor fluorescence and therefore demand an effective method to extract tumor signal from the strong background.

Different methods for background reduction have been proposed for *in vivo* FMI/FMT imaging (20–25). For example, tissue autofluorescence can be subtracted by exciting the endogenous fluorophores using blue-shifted excitation light (20–22), assuming that the excitation spectrum of the background fluorophores is much broader than that of the tumor-targeted fluorophores and the spatial distribution of autofluorescence remains the same within this broad excitation spectrum. However, it is hard for these methods to subtract the background fluorescence due to the nonspecific nanoparticle uptake in healthy tissues. Dual-tracer method was proposed to subtract the background fluorescence emitting from nonspecific distribution, by subtracting the untargeted tracer fluorescence from the targeted tracer fluorescence (23). However, this method assumes the two tracers have the same vascular permeability, which may not be true due to the different surface modification between targeted and untargeted nanoparticles. In addition, the administration of both untargeted and targeted tracers will cause additional dose burden and imaging time. Ale and Soubert et al. proposed methods to subtract simulated homogeneous background fluorescence based on the distance between excitation sources and detectors (24, 25). But the simulated background cannot reflect the true optical heterogeneity, especially after nanoparticles are administrated. Furthermore, some regularization-based reconstruction algorithms were also investigated to mitigate the influences of background fluorescence on the FMT reconstruction (26, 27). But these algorithms only suitable for the scenarios wherein the background fluorescence is far weaker than the target fluorescence.

In *in vivo* imaging, owing to the thickness variation and the heterogeneous optical properties of the imaged small animals, the background fluorescence emanating from the homogenous baseline fluorophores exhibits large dynamic range, which incurs spurious information that complicates the tumor detection. Venugopal et al. proposed an adaptive wide-field fluorescence imaging strategy by iteratively adapting the spatial intensity distribution across the excitation pattern, to reduce the influence of the large dynamic range (28). If the above method is adopted to our FMT imaging system based on rotating-view scheme, the proposed structured illumination would require extra devices (such as digital micro-mirror system) and the pre-optimization of illumination pattern for each rotating view would result in extra time burden. In the present study, simply modelled as the multiplication of a weighting coefficient and the distribution of excitation light transmitting through tissues, the large-dynamic-range background fluorescence can be directly subtracted to reduce its influence on tumor detection. The subtraction weighting coefficient was calculated using linear minimum mean square error estimation. Subsequently, an adaptive mask-based strategy was implemented to selectively refine the background fluorescence subtraction. *In vivo* mice experiments (n=11) were implemented to validate the reliability and robustness of the proposed method with respect to sensitive tumor detection.

## 2 Materials and methods

### 2.1 CT/FMT imaging guided irradiation platform

**Figure 1:**
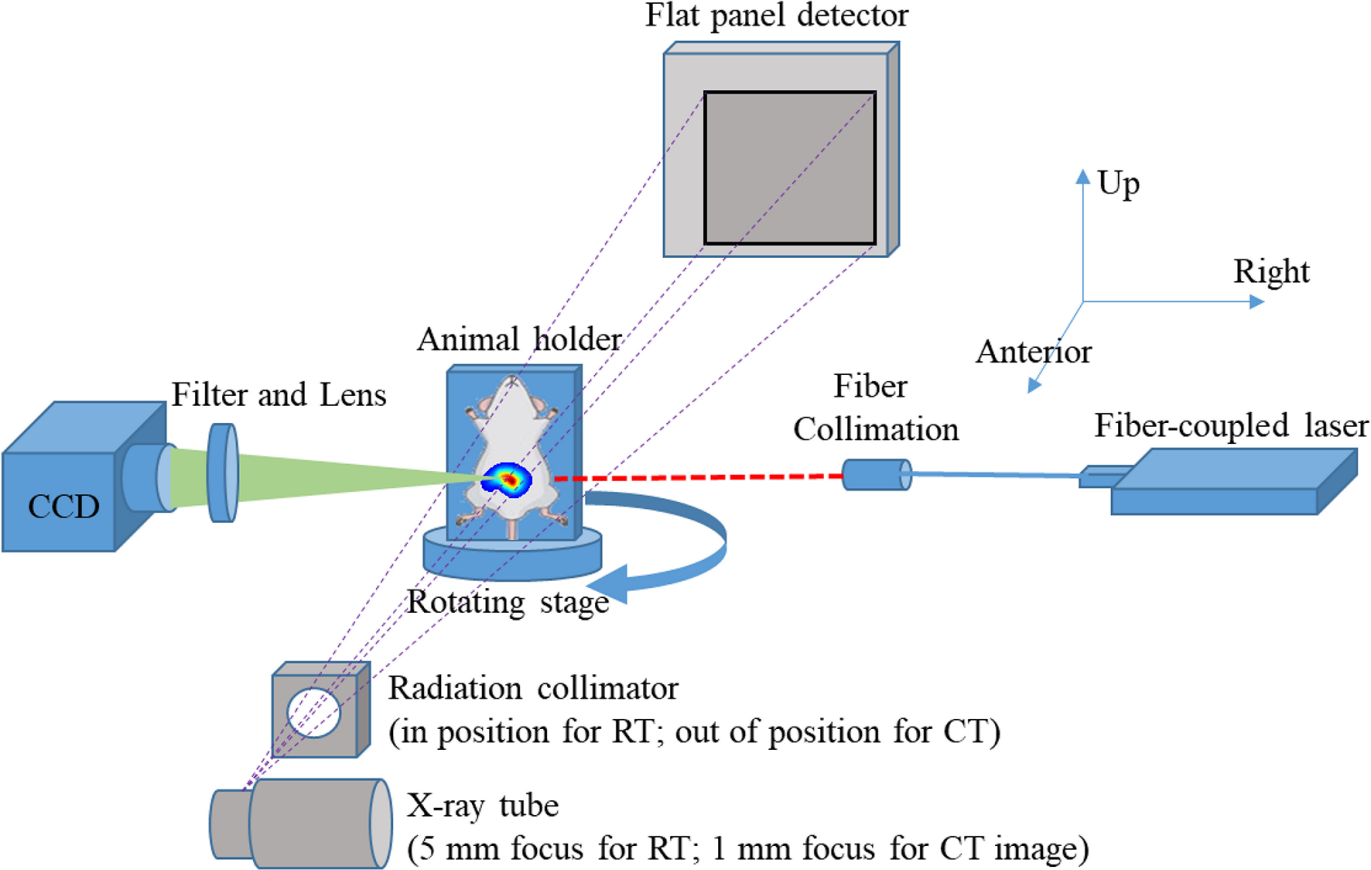
Schematic diagram of the multimodal CT/FMT imaging and image-guided irradiation platform.

**Figure 1** shows the schematic configuration of the in-house-developed small animal multimodality image radiation therapy platform (SAMMI-RT) consisting of X-ray CT, FMT, and radiation treatment. The X-ray tube (COMET AG, Flmatt, Switzerland) has 1 mm and 5 mm focal spots for cone beam CT and irradiation, respectively. For fluorescence imaging, a fiber-coupled laser (785 nm, full width at half maximum: 1 nm, output power: 20 mW) was collimated as a 2-mm-diameter light spot to excite the fluorescent nanoparticles in small animals. A neutral filter (OD=2, Thorlab, Newton, NJ, USA) and a fluorescence filter (832 nm, full width at half maximum: 37 nm, block-band OD>10, Semrock, Rochester, NY, USA) were installed in front of the CCD (quantum efficiency 95%, Andor, Belfast, Northern Ireland) to collect excitation and fluorescent images, respectively. A home-built geometrical calibration method was performed to rigorously register optical images onto CT coordinate (29). CT provides anatomical information used to construct three-dimensional mesh for FMT reconstruction. Previous study demonstrated that FMT could localize tumor and guide focal radiation delivery with submillimeter accuracy. More detail description about SAMMI-RT can be referred to (9, 30).

### 2.2 *In vivo* experiments

All the animal experiments were conducted under the protocols approved by the institutional animal care and use committee. We first conducted an *in vivo* experiment to demonstrate the reliability of our background subtraction method in a stringent situation, where the target fluorescence might be overlapped or overwhelmed with the strong fluorescence either from another target or from the background. To mimic this stringent situation for *in vivo* fluorescence imaging, a female athymic nude mouse (Harlan, Indianapolis, IN, USA) with orthotopic MDA-MB-231 breast tumor was intramurally injected with gold nanorods (GNRs, length: 38 nm, dimeter: 10 nm, excitation wavelength: 788 nm, emission wavelength: 808 nm, NanopartZ, Loveland, CO, USA). According to our experience based on several preliminary experiments, one hour post the intratumoral injection, a large majority of GNRs extravasated from the MDA-MB-231 breast tumor and accumulate in the bladder. In this scenario, the targets of interest are both tumor and bladder, however, the tumor fluorescence will be overwhelmed with the bladder fluorescence. To verify the accuracy of the proposed background subtraction method, after fluorescence imaging experiments on SAMMI-RT, this mouse was euthanized and dissected for organ fluorescence imaging on Kodak *In Vivo* Multispectral Imaging system (Carestream Health, New Haven, CT, USA).

**Figure 2:**
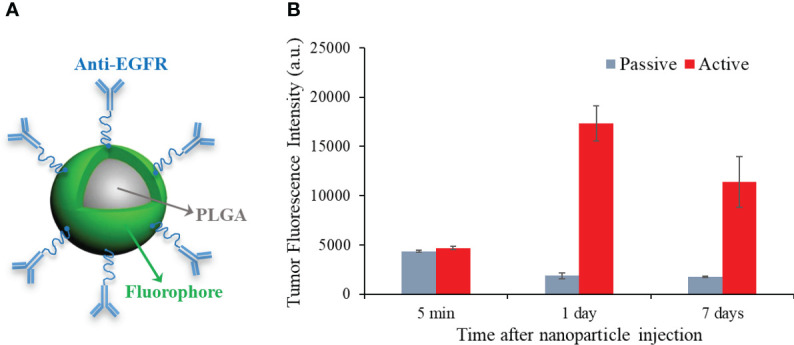
**(A)** Schematic structure of PLGA-anti-EGFR nanoparticles. **(B)** Longitudinal comparison of tumor accumulation for active (anti-EGFR) and passive targeting PLGA nanoparticles.

Then, we carried out *in vivo* active-targeting imaging experiments on white mice (BALB/c, Jackson laboratory, Bar Harbor, ME, USA). Compared to nude mice, white mice show additional hair-induced artifacts on fluorescence imaging. Therefore, white mice bearing breast tumors were implemented to further demonstrate the feasibility and robustness of our background subtraction method. To grow the orthotopic breast tumor on these immunocompetent mice, 5×10^5^ 4T1 cells were injected into the mammary fat pad of 4-week female white mice (n=10). Two weeks after tumor cell implantation, the fluorescent PLGA-anti-EGFR nanoparticles (300 µL, 10 mg/ml) were administrated *via* tail-vein injection after the mice were anesthetized with 2% isoflurane. **Figure 2A** shows the schematic structure of homemade PLGA-anti-EGFR nanoparticle, where the nanoparticle size is 262.9 ± 80.5 nm and the fluorescence excitation and emission wavelengths are 780 nm and 820 nm. **Figure 2B** compares the longitudinal tumor fluorescence intensity (on Kodak *In Vivo* Multispectral Imaging system) following the injection of active (anti-EGFR) and passive (IgG) targeting PLGA nanoparticles. The active targeting nanoparticles demonstrated enhanced tumor accumulation and long retention; peak appeared at 1 day after intravenous injection. Therefore, imaging experiments on SAMMI-RT were performed one day following PLGA-anti-EGFR injection. Then, the mouse was intravenously administered 200 µL iodinated contrast agent Iopamidal (1000 mg/kg body weight, Bracco Diagnostics, Princeton, NJ, USA) for contrast CT to delineate tumor. About 1 minute after Iopamidol injection, we began CT scan followed by fluorescence imaging. The white mice (n=10) were shaved around the abdominal region, before fluorescence imaging.

### 2.3 Background fluorescence subtraction algorithm

**Figure 3:**
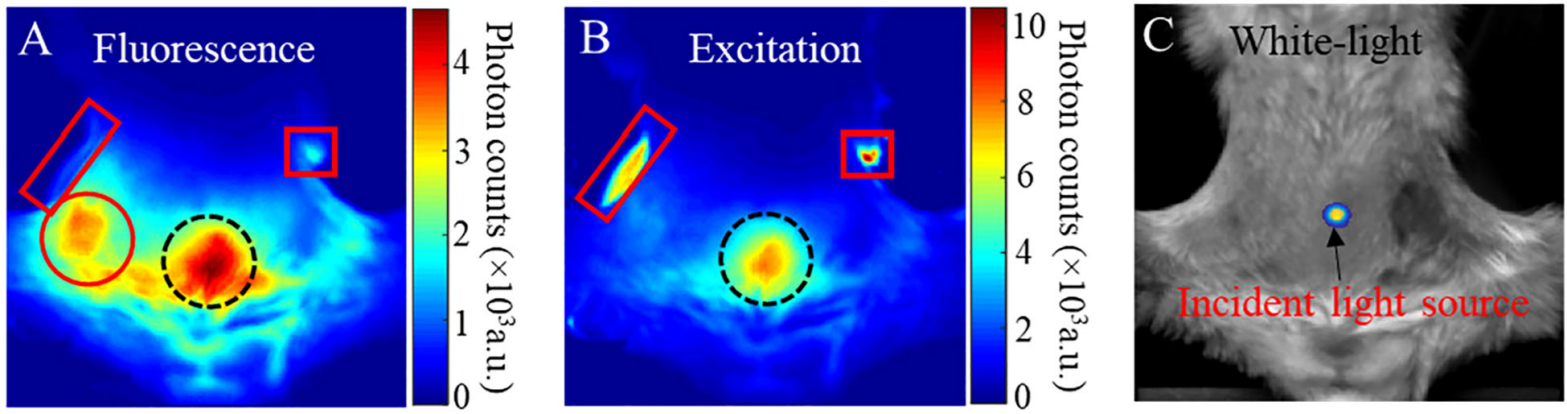
**(A)** Fluorescence image; red circle tumor signal, dotted circle gut uptake signal, left rectangle filter leakage animal holder signal, and right rectangle filter leakage mouse fur signal **(B)** Excitation image; Dotted circle gut excitation signal, right rectangle mouse fur reflection signal, left rectangle animal holder reflected signal. **(C)** white-light images of a mouse bearing a 4T1 tumor and received PLGA-anti-EGFR nanoparticle injection. The trans-illuminating point light source is overlapped onto **(C)**, to indicate the light-source position.

We take a fluorescence image in PLGA-anti-EGFR fluorescence imaging as a representative example to illustrate the necessity of background subtraction. Because contrast CT can delineate tumor, tumor region can be distinctly identified on the fluorescence image after geometric registration. As shown in **Figure 3A**, the fluorescence emanating from the tumor (left solid circle) is overwhelmed by the nonspecific signal from the gut (right dotted circle). Hence, it is difficult to directly identify tumor only from raw fluorescence image. The measured excitation distribution (shown in **Figure 3B**) exhibits large dynamic range, under the trans-illumination by a point source indicated in **Figure 3C**. The dotted circle region on **Figure 3A** was near the excitation source, therefore received much more excitation than the tumor area. In addition, the low absorption of the near-transparent bladder that localized around the dotted circle region may further enhance the transmission of light. Additionally, it is noteworthy that the two rectangle regions in **Figure 3B** correspond to the high-intensity excitation light reflecting from the residual hair (right rectangle) and the transparent animal holder (left rectangle), respectively. Although the fluorescence filter can highly attenuate the excitation light (OD >10), small fraction of excitation light still leaked through the fluorescence filter, as shown in the rectangle regions in **Figure 3A**. And in **Figure 3A**, the signal in the dotted circle region is much stronger than that in the rectangle region, which is opposite to the correspondent distribution in **Figure 3B**. This means that the signal in the dotted circle in **Figure 3A** was not caused by the excitation leakage, but mainly attributed to the fluorescence emitted by the nonspecific uptake of PLGA-anti-EGFR nanoparticles in the surrounding tissues.

We classify the internal fluorophores both exogenous and endogenous into two parts: baseline (homogeneous distribution), target (heterogeneous distribution). Therefore, the surface fluorescence signals *Flu*(*i, j*) can be defined as:


(1) $$\begin{array}{l} Flu\left(i,j\right)=Flu_{baseline}\left(i,j\right)+Flu_{target}\left(i,j\right)+Flu_{leak}\left(i,j\right)\\ i=1,\ldots ,L\;\;\;\;j=1,\ldots ,L \end{array}$$

Where *Flu_baseline_* and *Flu_target_* denotes the surface fluorescence from baseline and target fluorophores. *Flu_leak_* denotes the excitation leakage passing through fluorescence filter, (*i, j*) indicates the pixel position on CCD-measured image with matrix size L×L. The proposed method in this study is developed based on the fact that the near-infrared light in biological tissues at excitation and fluorescence wavelengths separated by short Stokes shift has similar optical characteristics and follows strongly correlated propagation patterns (31). Take adipose tissue for an example, the absorption (*μ_a_*) and reduced scattering (*μ’_s_*) coefficients are 0.029 cm^-1^ and 11.10 cm^-1^ as well as 0.031 cm^-1^ and 10.77 cm^-1^, calculated at the excitation (785 nm) and fluorescence (832 nm) wavelengths in this study.

Additionally, when exciting the fluorophores within the biological tissues, the generated fluorescence signal is linearly proportional to the excitation intensity and the internal fluorophore concentration. Therefore, the distribution of excitation field *Exc*(*i, j*) can be used to approximate the fluorescence distribution.


(2) $$Flu_{baseline}\left(i,j\right)=P_{baseline}\times Exc\left(i,j\right)$$

(3) $$Flu_{target}\left(i,j\right)=P_{target}\left(i,j\right)\times Exc\left(i,j\right)$$

where *Exc*(*i,j*) is the measurable excitation image. *P_baseline_* and *P_target_* (*i, j*) denote the weighting coefficients of baseline (i.e. homogenous distributed) and target (i.e. heterogenous distributed) fluorophores in the imaged animal. Because the baseline fluorophores are homogenously distributed, *P_baseline_* is position independent. In contrast, for the heterogeneously distributed target fluorophores, the value of *P_target_* depends on the position (*i,j*). Moreover, the excitation leakage part in fluorescence measurement can be written as:


(4) $$Flu_{leak}\left(i,j\right)=P_{leak}\times Exc\left(i,j\right)$$

where coefficient *P_leak_* accounts for the filter leakage ratio of the illumination light. Substituting Eqs. (2) and (4) to Eq. (1), we obtain:


(5) $$\begin{array}{l} Flu\left(i,j\right)=\left(P_{baseline}+P_{leak}\right)\times Exc\left(i,j\right)+Flu_{target}\left(i,j\right)\\ =P_{corr}\times Exc\left(i,j\right)+Flu_{target}\left(i,j\right) \end{array}$$

Equation (5) indicates that the “background fluorescence” induced by the homogenous baseline fluorophore and filter leakage, i.e. the first term on the right side of the Eq. (5), has the similar distribution pattern as excitation image. This fraction of fluorescence can be estimated by calculating the correlation between the collected fluorescence (*Flu*(*i, j*)) and excitation (*Exc*(*i, j*)) images, where *P_corr_* denotes the correlation coefficient. The linear minimum mean square error (LMMSE) estimation was adopted to calculate *P_corr_*:


(6) $$P_{corr}=\underset{P_{corr}}{\text{argmin}}\;{║Flu_{1\times L^{2}}-P_{corr}\times Exc_{1\times L^{2}}║}_{2}^{2}$$

Therefore, Eq. (6) is used to maximize background fluorescence, thus extracting the target fluorescence that is overwhelmed with the background fluorescence. The scalar *P_corr_* is derived by:


(7) $$P_{corr}=\left(Flu_{1\times L^{2}}\times Exc_{L^{2}\times 1}^{T}\right)/\left(Exc_{1\times L^{2}}\times Exc_{L^{2}\times 1}^{T}\right)$$

Because *P_corr_* is position independent, LMMSE is expressed in the form of vectors, where *Flu*_1×L_^2^ and *Exc_1×L_^2^* denote the reshaped 1-dimension vectors of the measured 2-dimension fluorescence image *Flu_L×L_* and excitation image *Exc_L×L_* $Exc_{L^{2}\times 1}^{T}$ denotes the transposition of vector *Exc_1×L_^2^*. Finally, the corrected target fluorescence distribution $Flu_{target}^{corr}$ is calculated as:


(8) $$\{\begin{array}{c} Flu_{target}^{corr}=Flu-P_{corr}\times Exc\\ Flu_{target}^{corr}\left(i,j\right)=0\;\;if\;\;Flu_{target}^{corr}\left(i,j\right)<0 \end{array}$$

**Figure 4:**
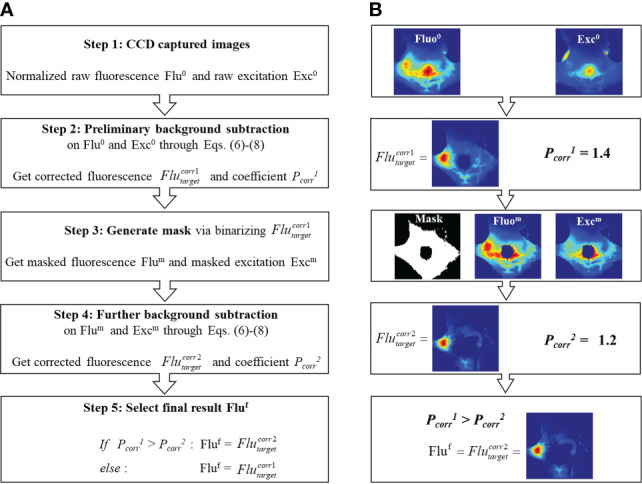
**(A)** Workflow of the adaptive mask-based background subtraction strategy. **(B)** Representative example illustrating how to extract tumor fluorescence from background fluorescence based on workflow **(A)**.

It should be noted that the background subtraction through Eq. (6) is based on the distribution of measured fluorescence and excitation fields which have different scales of signal intensity. Therefore, before using Eq. (7), the fluorescence and excitation images are respectively normalized to their own maximum. As shown in **Figure 3B**, the reflection artifact induced by mouse fur corresponds to the maximum of the excitation image. Therefore, the normalization to this high-intensity artifact (i.e., right square region in **Figure 3B**) can severely impact the true distribution of excitation photons transmitting though tissues. To eliminate the effect of reflection artifacts on the normalization and optimally subtract the background fluorescence, an Adaptive Mask-based Background Subtraction (AMBS) strategy is further implemented in a workflow as shown in **Figure 4**.

In the first step, based on the normalized raw fluorescence and excitation images, preliminary background subtraction is conducted through Eqs. (6)-(8) to get the subtraction coefficient $P_{corr}^{1}$ and the corrected fluorescence distribution $Flu_{target}^{corr1}$ . In steps 2 and 3, a mask is generated by binarizing the first-round correction $Flu_{target}^{corr1}$ and only keeping pixels with non-zero values. As is shown in the Step 3 of **Figure 4B**, with the use of mask, the reflection artifacts and high-intensity background region can be filtered out. Compared with the original excitation image (Step 1 in **Figure 4B**), the masked excitation image (Step 3 in **Figure 4B**) exhibits more detailed transmission field on the animal surface, rendering an optimized background subtraction. In step 4, a further background subtraction is conducted based on the masked fluorescence and excitation images, to get a new coefficient $P_{corr\;}^{2}$ and corrected fluorescence distribution $Flu_{target\;}^{corr2}$ . In step 5, we select the final residual fluorescence from $Flu_{target}^{corr1}$ and $Flu_{target}^{corr2}$ . As shown in Eq. (5), the subtraction coefficient *P_corr_* denotes the percentage of background in the fluorescence measurement. To avoid over-subtraction, we choose the corrected image with a smaller *P_corr_*.

### 2.4 FMT reconstruction

Coupled diffusion equation with Robin-type boundary condition was employed to model the propagation of near-infrared light in highly scattering media (32). The surface fluorescence measurement ${\text{Φ}}^{flu}\left(\vec{r_{d}},\vec{r_{s}}\right)$ detected at position $\vec{r_{d}}$ due to excitation spot located at $\vec{r_{s}}$ can be written as (33):


(9) $${\text{Φ}}^{flu}\left(\vec{r_{d}},\vec{r_{s}}\right)=\frac{vS^{flu}}{D^{flu}}\int^{}d^{3}\vec{r_{1}}G_{0}^{flu}\left(\vec{r_{d}},\vec{r_{1}}\right)x\left(\vec{r_{1}}\right)G_{0}^{exc}\left(\vec{r_{1}},\vec{r_{s}}\right)$$

where Green’s function $G_{0}^{exc}\left(\vec{r_{1}},\vec{r_{s}}\right)$ describes the propagation of excitation light from a source to a point $\vec{r_{1}}$ in the medium. Similarly, Green’s function $G_{0}^{flu}\left(\vec{r_{d}},\vec{r_{1}}\right)$ denotes the propagation of fluorescence light emanating from fluorophore at $\vec{r_{1}}$ to a detector $\vec{r_{d}}$ . Calibration factor *S* ^*flu*^ accounts for the unknown gain and attenuation factors of the optical system. *v* is the speed of light in the medium. *D* ^*flu*^ is the diffusion coefficient of medium at fluorescence wavelength. After discretizing the imaging domain generating from the 3D CT surface, the FMT reconstruction problem can be formulated as the following matrix equation (34, 35):


(10) $${\text{Φ}}^{flu}=WX$$

where *W* denotes the weight matrix mapping the relationship between the 2D fluorescence measurement Φ^*flu*^ and the 3D internal fluorophore distribution . The discrete cosine transform based L1-norm regularization algorithm was employed to solve the Eq. (10) for FMT reconstruction, and its detailed description can be referred to (36). Raw and AMBS corrected fluorescence images were respectively employed in Eq. (10), to investigate the performance of AMBS in FMT reconstruction improvement.

### 2.5 Evaluation index

To evaluate the performance of the proposed method, we introduce the tumor-to-background ratio (*TBR*) based on the profile traversing tumor center on the 2D fluorescence image. The *TBR* indicates the contrast of the mean fluorescence intensity inside (*mFlu^in^*) and outside (*mFlu^out^*) of the tumor region on the profile:


(11) $$TBR=mFlu^{in}/mFlu^{out}$$

Tumor center offset (*TCO*) and tumor volume overestimation (*TVO*) were used to quantitatively evaluate the performance of FMT respectively reconstructed using raw and background-corrected fluorescence images. *TCO* indicates the Euclidean distance between the tumor center (*x, y, z*) recovered in the FMT and the true center (*x_0_, y_0_, z_0_*) obtained from the contrast CT:


(12) $$TCO={\left[{\left(x-x_{0}\right)}^{2}+{\left(y-y_{0}\right)}^{2}+{\left(z-z_{0}\right)}^{2}\right]}^{1/2}$$

*TVO* denotes the percentage of over-estimated tumor volume in FMT (*V_FMT_-V_CT_*) relative to the true tumor volume *V_CT_*:


(13) $$TVO=\left(V_{FMT}-V_{CT}\right)/V_{CT}$$

## 3 Results

### 3.1 Reliability test

**Figure 5:**
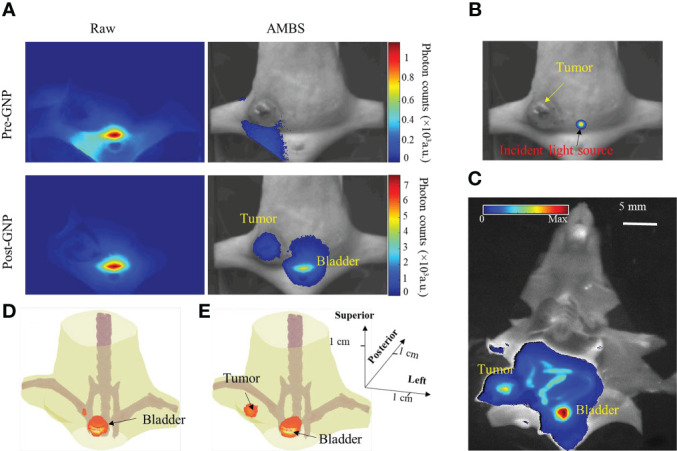
**(A)** Raw and AMBS-corrected fluorescence images before and after intratumoral injection of fluorescent GNRs. AMBS images were normalized to the maximum of the corresponding raw images. **(B)** Trans-illuminating light source is overlapped onto white-light image to indicate the excitation location. **(C)** Organ fluorescence imaging of the dissected animal, using Kodak *In Vivo* Multispectral Imaging system. **(D, E)** FMT reconstruction based on the raw and AMBS-corrected fluorescence images, respectively.

It’s noteworthy that, in the example (**Figure 3**), the strong background fluorescence originated from the regions which have low absorption characteristic and locate near the excitation light source. If target of interest resides in these regions, our method can still effectively suppress the background fluorescence and extract the target signal, which is illustrated through the following GNR fluorescence imaging experiment. **Figure 5A** shows the raw and AMBS-corrected fluorescence images, before and after intertumoral GNR injection. The excitation location is indicated on **Figure 5B**. Owing to the low absorption of the bladder, more autofluorescence and possibly unfiltered excitation light will penetrate through this region. Without effective background subtraction, it is difficult to determine if there were GNRs in the tumor or bladder, since the fluorescence images appeared similar between the pre- and post-injection raw images (**Figure 5A**, column 1). And the tumor was not clearly visible in the raw post-GNR fluorescence image. Based on the AMBS correction, we can reliably identify if there exits GNRs in the imaged body. And by effectively subtracting the background fluorescence, AMBS clearly extracted the GNR fluorescence emanating from both tumor and bladder (**Figure 5A**, column 2), which can be directly validated through the organ fluorescence image acquired on Kodak *In Vivo* multispectral Imaging System (**Figure 5C**). Additionally, the highlighted GNR fluorescence in tumor and bladder, as shown in **Figure 5C**, can also be treated as the benchmark to validate the accuracy of FMT reconstruction. **Figure 5D** shows that FMT reconstruction using the raw fluorescence images can only recover the GNRs accumulated in the bladder, due to the overly strong bladder fluorescence signal. In contrast, FMT reconstruction using AMBS-corrected fluorescence images recovered the GNRs accumulated in both the breast tumor and bladder (**Figure 5E**), bolstering that our proposed background subtraction strategy can improve the reliability of FMT, even when the target signal is overwhelmed by strong background.

### 3.2 Robustness test

**Figure 6:**
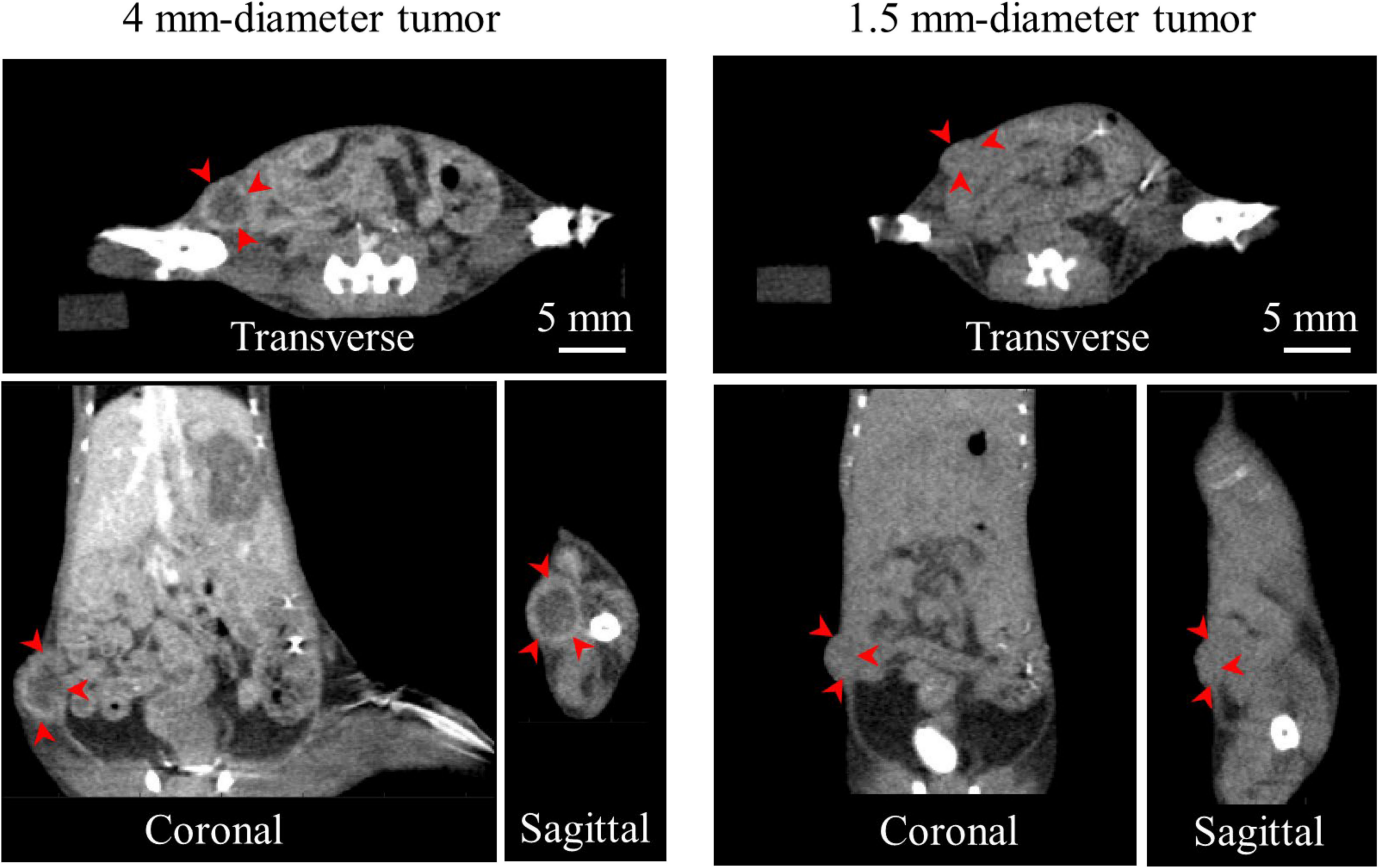
Contrast CT slices of 2 (out of 10) mice bearing orthotopic breast tumors of different sizes, in three orthogonal views. Arrows indicate the contrast enhanced tumor edge.

As an example, **Figure 6** shows the contrast CT slices of 2 (out of 10) mice bearing breast tumors of different sizes. The large tumor (4 mm diameter) was clearly delineated through the enhanced tumor edge. The small tumor (1.5 mm), however, demonstrated minimal contrast enhancement, and was identified only from its morphological appearance. In this study, the tumor contoured from contrast CT was treated as the benchmark to verify the accuracy of AMBS in tumor identification.

**Figure 7:**
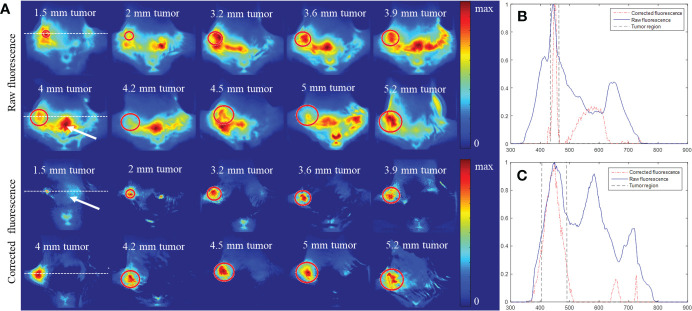
Comparison of fluorescence distribution before and after background subtraction for 10 mice bearing 4T1 breast tumors of different sizes. All the fluorescence images were obtained 1 day after intravenous injection of PLGA-anti-EGFR nanoparticles. **(A)** Raw and background-corrected fluorescence images, which were normalized by their own maximum. The circle marks the tumor differentiated from contrast CT. **(B, C)** Profiles across the 1.5 mm and 4 mm tumor, respectively (along the dotted lines in the 1st column of **(A)**). All the profiles were normalized to their own maximum.

**Figure 7A** shows the raw and AMBS-corrected fluorescence images for the 10 white mice bearing 4T1 tumors of different sizes (top half: ≥ 4mm; bottom half:< 4mm). The first column shows the fluorescence images of the mice bearing 4-mm and 1.5-mm tumors (as indicated in **Figure 6**). Since the optical and CT coordinates are geometrically registered, the true tumor location shown in CT can be precisely projected onto the fluorescence images. The red circles on **Figure 7** indicate the tumor region acquired from CT. As shown in the raw fluorescence images, the tumor fluorescence (circle region) mixes up with the nonspecific background fluorescence with large dynamic range, complicating the tumor detection. In contrast, all the tumor fluorescence signals were effectively extracted in the corrected fluorescence, validating the feasibility and robustness of the proposed AMBS method. Compared with the raw fluorescence images, corrected fluorescence images show significantly higher tumor-to-background contrast, which is beneficial to early-stage small tumor detection. As examples, **Figures 7B, C** show the normalized profiles plotted along the dotted lines (shown in **Figure 7A**, column 1) across the tumor regions. The profiles quantitatively demonstrate that the strong artifacts (indicated by the signal peaks) outside of the tumor region is effectively suppressed. In addition, the tumor sizes in the corrected fluorescence images are much closer to the truth than that in the raw fluorescence images.

**Figure 8:**
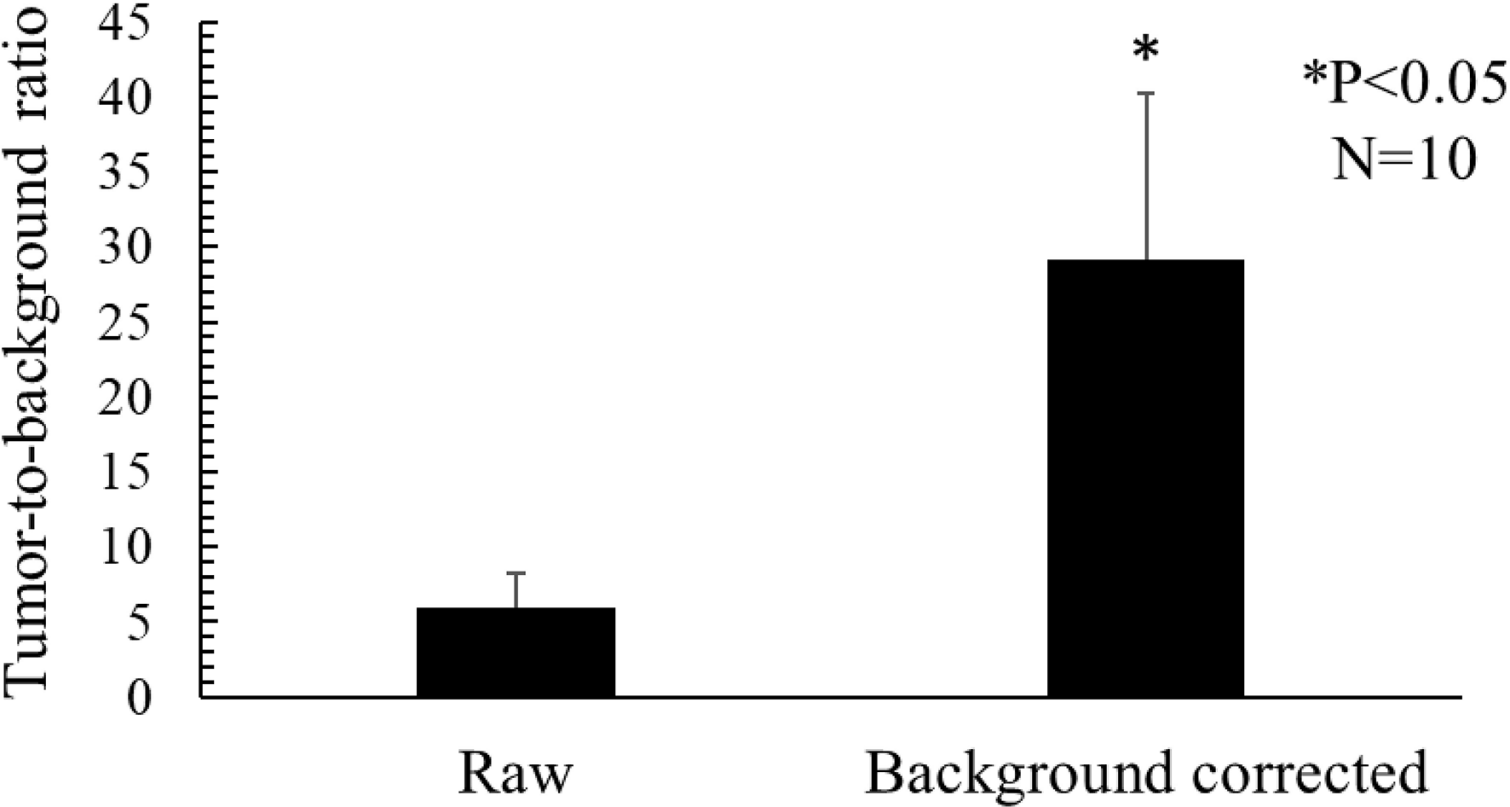
Mean tumor-to-background ratios for the raw and background corrected fluorescence images.

To further quantitatively demonstrate the effectiveness of the developed approach, the tumor-to-background ratios for all the 10 experiments were calculated from the profiles traversing the tumor centers in **Figure 7A**, based on Eq. (11). Student’s t-test analysis result in **Figure 8** suggests that the proposed strategy significantly (P<0.05, N=10) improved the tumor-to-background contrast.

### 3.3 Active-targeting FMT tumor recovery

**Figure 9:**
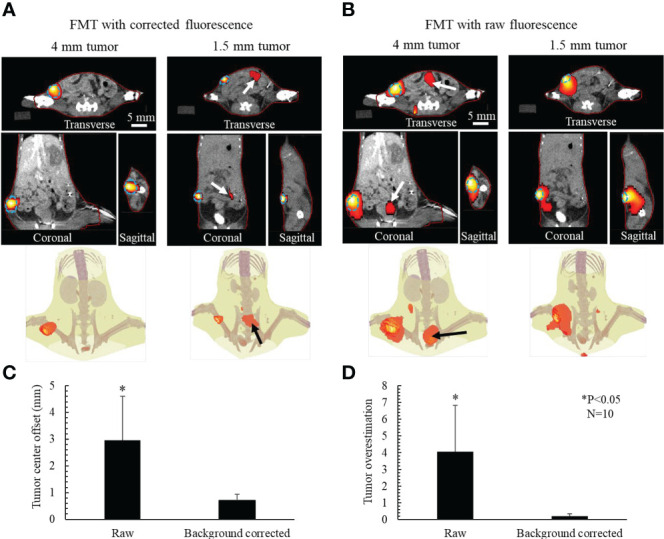
Active-targeting FMT reconstruction respectively based on AMBS-corrected **(A)** and raw **(B)** fluorescence, for 2 (out of 10) mice bearing breast tumors with different sizes. Blue dotted line delineates the tumor edge obtained from CT. FMT (red colorwash) is superimposed on CT. Red line denotes the mouse contour used in FMT reconstruction. Bottom rows in **(A, B)** show the 3D rendering of FMT in the CT bony anatomy. Arrows in **(A)** point to the reconstructed residual uptake in gut. Arrows in **(B)** point to the artifacts caused by the nonspecific fluorophore update in abdomen. **(C)** Mean tumor center offset and **(D)** tumor volume overestimation for the 10 mice experiments.

**Figure 9A** shows the 3D FMT results reconstructed using the AMBS-corrected fluorescence images, for the same example mice shown in **Figure 6**. The FMT and CT coordinates are physically registered in our system through rigorously geometrical calibration (29). The red line denotes the mouse contour used for FMT reconstruction. The match between the FMT contour and CT animal surface confirmed the accuracy of our geometrical calibration. The dotted line delineates the tumor edge obtained from contrast CT. A good coincidence of the tumor localization and tumor volume was observed between FMT and CT in all three orthogonal views, indicating that FMT with AMBS-corrected fluorescence images can accurately recovery the tumors even for the small tumor. The last row in **Figure 9A** displays the 3D rendering of FMT reconstruction fused with the CT bony anatomy. **Figure 9B** shows the FMT results reconstructed using the raw fluorescence images. Because the tumor fluorescence is surrounded by the strong background fluorescence (as shown in the raw fluorescence images in **Figure 7**), the FMT tumor volume in **Figure 9B** is much larger than the truth, especially for the small 1.5 mm tumor. And the high-intensity fluorescence in the bladder region (as shown in **Figure 7**, raw 2D fluorescence image of 4-mm tumor) induced the false-positive 3D-FMT target recovery in bladder (marked as arrows in **Figure 9B**).

**Figures 9C, D** illustrate the comparison between the FMT respectively reconstructed from corrected and raw fluorescence images, in terms of tumor center offsets and tumor volume overestimation. After background subtraction, the accuracy of FMT reconstruction regarding both tumor localization and tumor volume was significantly improved (P<0.05, N=10).

## 4 Discussion

In this work, we studied the improvement of tumor detection accuracy by subtracting nonspecific background in the active-targeting fluorescence imaging. We classify the internal fluorophores, including both the exogenous and endogenous fluorophores, into baseline (homogeneously distributed) and target (heterogeneously distributed) parts. Due to the thickness variation and the heterogeneous optical properties of the imaged subject, the baseline fluorophores generate highly spatial varying fluorescence, which induces artifacts in the 2D fluorescence imaging and 3D FMT reconstruction and complicates tumor detection. Due to the short Stokes shift, this background fluorescence was linearly estimated from the distribution of excitation light propagating through tissues. In addition, for the active-targeting imaging, the fluorescence signal emanating from the tumor-targeted fluorophores is almost sparse relative to the entire background. Based on these facts, we employ the linear minimum mean square error estimation (i.e. Eq. (6)) to optimally subtract the background fluorescence. The robustness of our method in tumor fluorescence extraction has been validated through PLGA-anti-EGFR fluorescence imaging experiments on 10 mice (as shown in **Figure 7**). It is noteworthy that the targets in 10 PLGA-anti-EGFR experiments are located relatively far away from the excitation location. To verify the capability of our method under more stringent situation, i.e. target of interest resides near the excitation location, we also conducted a GNR fluorescence imaging experiment. GNR experiment demonstrated that our method can reliably extract out the target fluorescence, even though there is serious overlap between background and target fluorescence. In the present study, the background subtraction is based on the distribution (rather than the absolute intensity) of fluorescence and excitation light measurements. This means the CCD captured fluorescence and excitation images were required to be normalized to their own maximum, before background subtraction. Thus, as long as the relative distribution of excitation field keeps unchanged (i.e. there is no saturated region), the incident light intensity will not affect the estimated background fluorescence distribution. Although high-performance fluorescence bandpass filter was adopted, a small fraction of excitation light might leak through, inducing false fluorescence signal. By introducing leakage coefficient (implicitly included in as shown in Eqs. (4-5)) in our proposed adaptive mask-based background subtraction strategy, these parts of false fluorescence signal could also be eliminated.

FMT has been widely applied to small animal research for *in vivo* monitoring molecular and cellular process, by exploiting the specificity of fluorescent biomarkers. The application of FMT in human, however, is limited, owing to the short penetration depth of light and small amount of FDA approved fluorescent agents (37). Nonetheless, benefiting from its high sensitivity, FMT still get attention in clinical diagnosis, such as synovitis imaging of finger and breast cancer detection (38, 39). Moreover, because of PLGA biodegradability and biocompatibility it been widely chosen to design nanoparticles as drug delivery systems in cancer therapy (40). Combining active-targeting technique, FMT is a good choice to *in vivo* monitor the PLGA-based drug delivery. In this study, to benchmark against the FMT reconstruction, we employed contrast CT. Generally, only a limited number of tumor models can be differentiated in contrast CT. For example, poorly vascularized tumor models cannot be detected with it. And as shown in our results (**Figure 6**), even though to the high-vascularized tumor model, contrast CT cannot delineate small tumor accumulated with minimal contrast agents. By comparison, using actively targeting fluorescence probes, FMT can detect tumors with a higher sensitivity and specificity. Plus, the fluorescence intensity can reflect molecular and cellular activities of tumors, thereby monitoring the tumor growth and treatment response in molecular level. Previous studies have demonstrated that fluorescence imaging can offer comparable performance to PET imaging, in metabolically assessing tumor treatment (41).

The present primarily focuses on how to improve FMT imaging quality by pre-processing collected surface fluorescence images. The FMT and CT imaging modalities on SAMMI-RT are intrinsically co-registered in the same coordinate system, affording fusing reconstructed fluorescence distributions with morphological features. More importantly, the CT images, automatically segmented into different organ and tissue segments can be used to improve the quality of FMT reconstruction, by providing structural priors for FMT reconstruction inverse problem (42, 43). More studies will be conducted to investigate the synergetic FMT reconstruction by combing AMBS background subtraction with CT-based structural priors. Furthermore, although iodine-contrast CT delineated the tumor, the histopathological details are missed. A whole-mouse cryosection imaging technique (or cryo-imaging) demonstrates the capability to provide more detailed contrast not obtained with traditional gray-scale medical imaging modalities (CT, MRI, etc.) (44, 45). Visualization of color anatomy and molecular fluorescence in whole-mouse cryo-imaging will provide more histopathological information to benchmark against the fidelity of background fluorescence subtraction. Compared to other *in vivo* imaging modalities, fluorescence imaging exhibits its distinct ability to employ multiple imaging channels (46). For example, to assess the treatment response in different histopathological subsets of tumors, multiple fluorescent nanoparticles targeting to different biomarkers will be used. The proposed background fluorescence subtraction method can be directly extended to this *in vivo* multi-channel fluorescence imaging, because the large optical imaging window from ∼600 to 1000 nm enables the use of multiple fluorescent probes simultaneously without significant bleed through among the imaging channels.

The goal of the present study focuses on how to effectively identify tumor in the situation wherein background fluorescence severely interferes target signal. To further evaluate the quantitative accuracy of our method, efforts are needed to study the correlation between corrected fluorescence and true fluorescence in a more controllable way through *ad hoc* phantom calibration experiments. Additionally, the background subtraction strategy proposed here is only used to subtract the fluorescence emanating from the homogeneous part of endogenous fluorophores and exogenous nonspecifically-uptaken nanoparticles. Therefore, the signal from the heterogeneous nonspecifically-uptaken nanoparticles ingested by organs other than the tumors will be reserved in the corrected fluorescence image and in the eventual FMT reconstruction. As an instance, the intertumoral GNR imaging experiment confirmed that the nonspecific uptake by bladder was indeed reserved in the corrected fluorescence images, and both the tumor and GNR-filled bladder were reliably reconstructed in FMT. To further eliminate the fluorescence emitted by the non-tumor tissues, the prior spatial distribution of the nonspecifically-accumulated nanoparticles is needed, which can be realized through dual-tracer method, where the passively-targeted nanoparticles should be designed to possess the similar structure (e.g. size, shape, etc.) as the actively-targeted nanoparticles (23). It is worth mentioning that a point-excitation light source was used for FMT imaging in the present study. For the whole-body metastasis study, large-area light source or raster-scanned source is needed to excite the imaging object. Notwithstanding, our method is still expected to be valid, provided there exists no over-exposure region in the excitation image.

## 5 Conclusion

In conclusion, the proposed adaptive background subtraction method improves the accuracy and reliability of fluorescence imaging in tumor detection and leads to accurate tumor localization with high target-to-background contrast. It can find broad applications not only in 3D fluorescence tomography, but also more importantly in 2D planar fluorescence imaging which has high throughout and is widely adopted in biology research. This method will particularly help early tumor detection and fluorescence imaging guided radiotherapy. It can also benefit translational nanoparticle research where fluorescence imaging is heavily utilized.

## Data availability statement

The original contributions presented in the study are included in the article/supplementary material. Further inquiries can be directed to the corresponding authors.

## Ethics statement

The animal study was reviewed and approved by Institutional Animal Care and Use Committee Office (IACUC), University of Miami.

## Author contributions

Conceptualization, JS and JF; methodology, JV and DH, RS, MA, JS, and JF; validation, JV, RS, DH, JF, and JS; formal analysis, JS and DH; investigation, JV, RS, DH, JS, WT, AP, and JF; resources, JS, AP, JF, and ND.; data acquisition, RS, DH, and JS; writing—original draft preparation, JV and JS; writing—review and editing, JS and JF; supervision, JF and JS; funding acquisition, AP and JS. All authors contributed to the article and approved the submitted version.
